# Relationship between the perceived strength of countries’ primary care system and COVID-19 mortality: an international survey study

**DOI:** 10.3399/bjgpopen20X101129

**Published:** 2020-09-09

**Authors:** Felicity Goodyear-Smith, Karen Kinder, Cristina Mannie, Stefan Strydom, Andrew Bazemore, Robert L Phillips

**Affiliations:** 1 Department of General Practice & Primary Health Care, University of Auckland, Auckland, New Zealand; 2 Fachgebiet Management im Gesundheitswesen, Technische Universität Berlin, Berlin, Germany; 3 American Board of Family Medicine, Washington, DC, US

**Keywords:** Primary health care, COVID-19, Mortality rate

## Abstract

**Background:**

Strong primary health care (PHC) is the cornerstone for universal health coverage and a country’s health emergency response. PHC includes public health and first-contact primary care (PC). Internationally, the spread of COVID-19 and mortality rates vary widely. The authors hypothesised that countries perceived to have strong PHC have lower COVID-19 mortality rates.

**Aim:**

To compare perceptions of PC experts on PC system strength, pandemic preparedness, and response with COVID-19 mortality rates in countries globally.

**Design & setting:**

A convenience sample of international PHC experts (clinicians, researchers, and policymakers) completed an online survey (in English or Spanish) on country-level PC attributes and pandemic responses.

**Method:**

Analyses of perceived PC strength, pandemic plan use, border controls, movement restriction, and testing against COVID-19 mortality were undertaken for 38 countries with ≥5 responses.

**Results:**

In total, 1035 responses were received from 111 countries, with 1 to 163 responders per country. The 38 countries with ≥5 responses were included in the analyses. All world regions and economic tiers were represented. No correlation was found between PC strength and mortality. Country-level mortality negatively correlated with perceived stringent border control, movement restriction, and testing regimes.

**Conclusion:**

Countries perceived by expert participants as having a prepared pandemic plan and a strong PC system did not necessarily experience lower COVID-19 mortality rates. What appears to make a difference to containment is if and when the plan is implemented, and how PHC is mobilised to respond. Many factors contribute to spread and outcomes. Important responses are first to limit COVID-19 entry across borders, then to mobilise PHC, integrating the public health and PC sectors to mitigate spread and reduce burden on hospitals through hygiene, physical distancing, testing, triaging, and contract-tracing measures.

## How this fits in

Having a strong PHC approach is the cornerstone for universal health coverage and the foundation of any country’s health emergency response. Countries perceived to have strong PC systems are expected to have lower mortality rates from COVID-19. However, from the perspectives of PC experts, having a prepared pandemic plan and strong PC are insufficient. Important responses are first to limit COVID-19 entry across borders, then to mobilise PHC, integrating the public health and PC sectors to mitigate spread and reduce burden on hospitals through hygiene, physical distancing, testing, triaging, and contract-tracing measures.

## Introduction

As the COVID-19 pandemic has swept the world, individual countries have responded differently to the threat and have experienced vastly different results with respect to the human cost of disease and mortality.

There are effectively three main thresholds to managing a pandemic. The first is to mitigate entry of the infection into the country. This requires stopping the incoming movement of people from abroad, or for those who do enter, such as returning residents, imposing strict quarantine until it is established that they are COVID-19 negative. Second, if the infection has been introduced, PHC measures can be activated to reduce the spread.^1^ These include traditional public health measures such as hand-washing, limiting person-to-person contact through physical distancing, self-isolation measures, and personal protective equipment, as well as testing, contract tracing, and surveillance. Third, where containment is ineffective and there is increasing community spread, reduction in the rate of mortality from the disease requires hospitalisation for severe cases, with provision of supportive measures including oxygenation, intensive care, and ventilation.

There are a number of ways PHC can help reduce the spread of COVID-19. A PHC approach includes public health, PC, and community-based social services.^2^ The role of public health includes rapid and broad population testing, contact tracing, and surveillance of cases, as well as communicating consistent personal prevention messages such as the promotion of healthy behaviours (for example, hand-washing), and avoiding close contact with potentially infected people.

Building on the 1978 Alma-Ata Declaration,^3^ the Astana Declaration of 2018^4^ asserts that strong PHC is the cornerstone for universal health coverage, and the most inclusive, effective, and efficient approach to enhance people’s physical and mental health, and social wellbeing. The Declaration calls for an *’* *accessible, equitable, safe, of high quality, comprehensive, efficient, acceptable, available and affordable* *’* approach delivering continuous, integrated people-centred services.^4^ Further, Barbara Starfield identified that high performing PC provides access to first contact, patient-centred care that is comprehensive, continuous over time while coordinating services.^5^

According to Dunlop *et al*, strong PC systems form the foundation of any emergency response.^6^ Community-based practitioners provide the vast majority of health care across the spectrum of prevention, preparedness, response, and recovery, and hence should be a vital component of an effective pandemic response. PC can play a role in conducting community-based testing, triaging cases, and providing first-contact care, referring only those cases that exacerbate to a severity requiring hospital care. Flexibility, adaption, and innovation are needed, with possible workforce mobilisation, task-shifting, and provision of electronic consultations where possible for ongoing non-COVID-19 comprehensive PC.

The aim of this study was to test the hypothesis that countries perceived to have stronger PC systems have lower mortality rates from COVID-19 than those with weaker PC systems.

## Method

### Setting and participants

This study is a natural experiment using a convenience sample. An original online anonymous questionnaire was developed to capture the variables of interest, administered via Qualtrics. Participants were international PC experts (clinicians, researchers, and policymakers). All global regions and economic tiers (low-, middle-, and high-income) were included. Invitations to participate were disseminated through a wide range of PC networks, augmented by a snowball technique.

The English version was launched 15 April 2020. Responding to considerable interest, the survey was translated into Spanish, which was available from 28 April 2020. Both language versions closed 4 May 2020.

Questions addressed the perceived nature of their country’s PC system, availability of a national pandemic plan, and various strategies employed in response to the pandemic (see Supplementary Appendix S1 for full survey).

### Response variable

This is defined as the maximum mortality rate for a country on a 7-day moving average basis. This measure aims to quantify the severity of the pandemic in a country at its worst point. Country-level data on mortality rates from COVID-19 were extracted from John Hopkins University of Medicine on 24 June 2020.^7^

### Measure of perceived PHC system strength

The survey asked about the availability of accessible, comprehensive care for all or the majority of the population, PC coordination and gatekeeping of specialist care, use of a unique patient identifier within the healthcare system, comprehensive patient records, and e-consultations prior to the pandemic. Affirmative responses were considered likely to be indicative of stronger PC systems.

### Analyses

Countries having similar characteristics were grouped, using K-means clustering, into three clusters (strong, moderate, or weak) suggestive of strength of existing healthcare systems (as described above). The relationship between PC system strength and mortality was explored by comparing the maximum mortality rates observed to date for each country. Bivariate analyses were performed to determine the correlation between PC system variables and with mortality. Spearman correlation coefficients (*ρ*) were calculated for pairs of variables.

A single PC strength metric was defined as the average response across the seven PC indicators captured (accessible, comprehensive, coordinated care, enrolled population, specialist gatekeeping, referral system, and multi-disciplinary team approach), given the positive correlations observed between the individual PC variables. Subgroup analyses were performed to control for country income classification^8^ and age profile: aged ≥65 years population below the median; above the median; or above the 75^th^ percentile.^9^

Individual participant responses were aggregated to country level to perform bivariate analysis with country-level outcomes. For each multiple choice answer, the country-level response was calculated as the proportion of responders from each country who selected each response. For numeric answers, the country-level response was calculated as the mean across all participants within each country. For ranking questions, the country-level response was calculated as the proportion of responders who ranked each choice as most important. Some multiple choice responses were grouped together to remove noise. Statistical analyses were performed using R (version 3.6.3).

## Results

### Participant characteristics

A total of 1131 survey responses from 114 countries were collected. Ninety-six uncompleted surveys were excluded, leaving 1035 responses from 111 countries. The number of responses from each country varied from 163 (Australia) to 34 countries with a single responder each (see Supplementary Table S1 and Supplementary Figure S1). Thirty-eight countries with ≥5 responses accounted for 87% (*n* = 897) of all responses. Countries from all world regions and economic tiers were represented. The majority of responders (57%) were female; 51% of total responders were aged between 30–49 years; 41% were aged between 50–69 years; 73% (*n* = 756) identified as PC clinicians, 17% (*n* = 175) as academics, 6% (*n* = 60) as policymakers, with the remainder (4%, *n* = 44) as ‘other’. The English version of the survey was completed by 92% (*n* = 954) of responders, and the Spanish version by 8% (*n* = 81).

### Relative mortality

**Figure 1.**
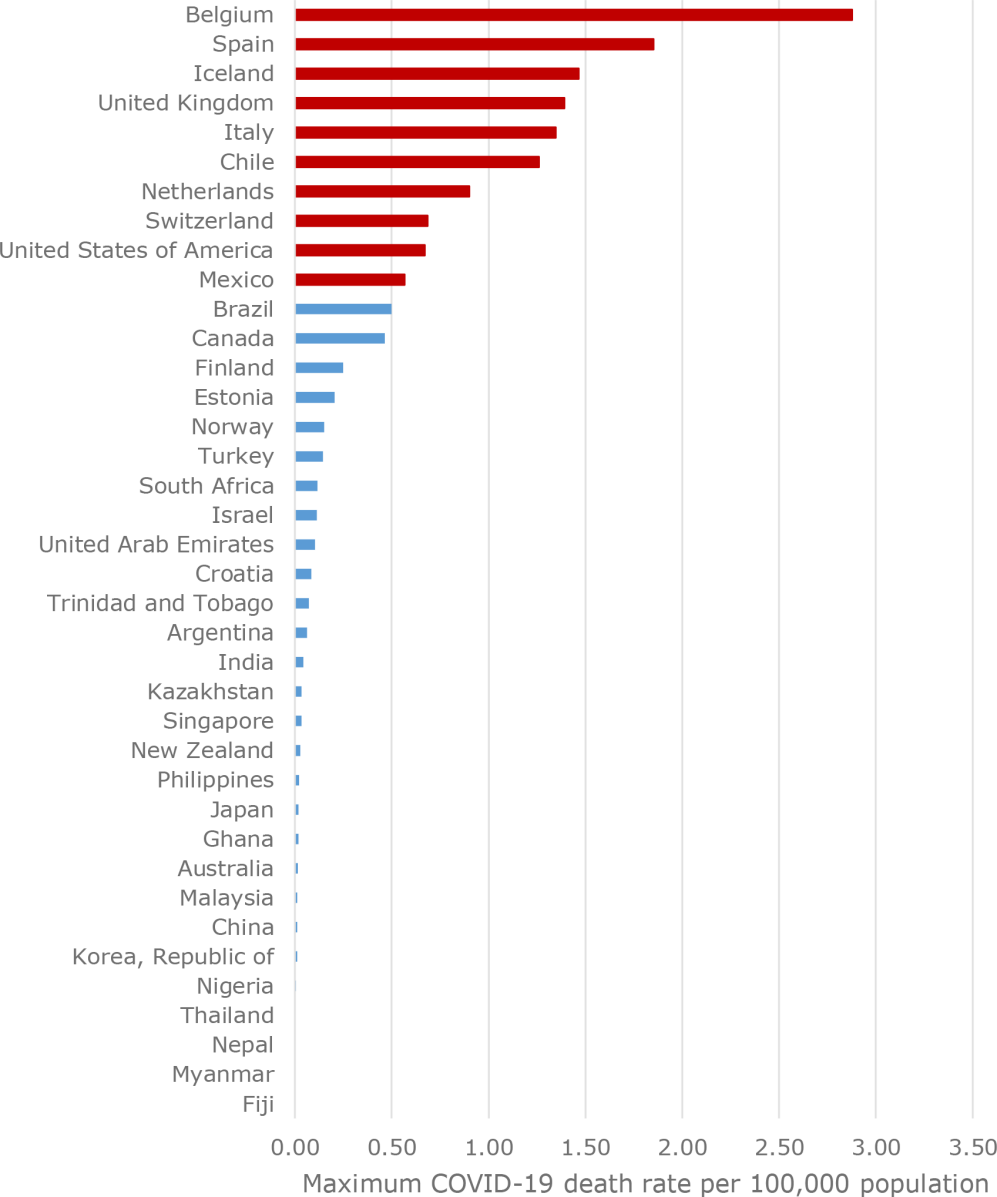
Maximum COVID-19 mortality rate by country

For the 38 countries, the maximum mortality rate (expressed per 100 000 population) ranged from 0.0 (Fiji) to 2.88 (Belgium) (Figure 1). The median maximum mortality rate across the 38 countries was 0.09. The countries highlighted in red were above the 75^th^ percentile in terms of maximum mortality rate for the 38 countries.

### Strength of PC system

**Figure 2.**
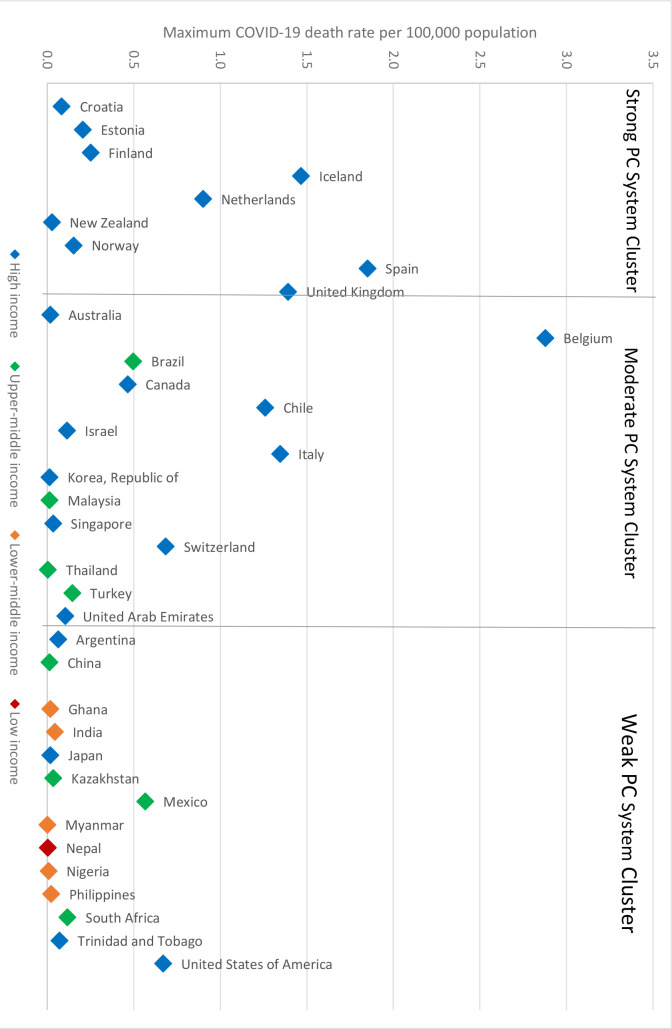
Comparison of PC system strength with maximum COVID-19 mortality rate (by country income)

For the 38 countries, responders’ perceptions of the strength of their country’s PC systems were categorised as strong, moderate, or weak (see Supplementary Table S2). There were positive correlations between all the individual PC variables (see Supplementary Figure S2). Particularly strong correlations exist between PC being available to the majority of the population and PC being comprehensive (*ρ* = 0.79), and between having a unique identifier across the population and having comprehensive electronic medical records (ρ = 0.79). Overall, there was no evidence that countries with strong PC systems experienced lower COVID-19-related mortality. To the contrary, some countries perceived by responders as having strong PC systems relative to other countries experienced higher mortality rates in general (Figure 2).

Subgroup analyses to control for older populations or income found no systematic evidence that countries with stronger PC systems were able to mitigate the severity of their COVID-19 pandemics to a better extent than countries with weaker PC systems (Table 1). High-income countries appeared to be most negatively impacted by COVID-19 mortalities.

**Table 1.** Country maximum COVID-19 mortality rates controlling for various confounders

**Subgroup^a,b,c,d^**	***n***	**Spearman’s** *ρ*	***P* value**
All countries	38	0.5275	0.0007
Countries with testing policy = 1	25	0.5297	0.0065
Countries with testing policy = 2	6	0.3714	0.4685
Countries with border closures = 0	7	0.6071	0.1482
Countries with border closures = 3	13	0.4622	0.1118
Countries with border closures = 4	9	0.5500	0.1250
Countries with young populations	19	0.4952	0.0311
Countries with older populations	19	0.1667	0.4953
Countries with oldest populations	10	0.0303	0.9338
High-income countries	23	0.2490	0.2519
Upper-middle income countries	9	0.2176	0.5739
Lower-middle income countries	5	–0.3591	0.5528

^a^Testing policy as ranked by the Oxford COVID-19 Government Response Tracker: 0 = no testing policy; 1 = only those who both a) have symptoms; and b) meet specific criteria (for example, key workers, admitted to hospital, came into contact with a known case, and/or returned from overseas); 2 = testing of anyone showing COVID-19 symptoms; 3 = open public testing (for example, ’drive through’ testing available to asymptomatic people); and Blank = no data. ^b^Border closure metric measures restrictions on international travel as ranked by the Oxford COVID-19 Government Response Tracker: 0 = no restrictions; 1 = screening arrivals; 2 = quarantine arrivals from some or all regions; 3 = ban arrivals from some regions; 4 = ban on all regions or total border closure; and Blank = no data. ^c^Age of population: young populations = proportion of the population aged ≥65 years is below 50^th^ percentile; older populations = proportion of the population aged ≥65 years is between 50^th^ and 75^th^ percentile; and oldest populations = proportion of the population aged ≥65 years is above the 75^th^ percentile. ^d^Only subgroups with a minimum of five countries were included, hence low-income countries were excluded from subgroup analyses.

### Perceptions of pandemic plan and execution

**Figure 3.**
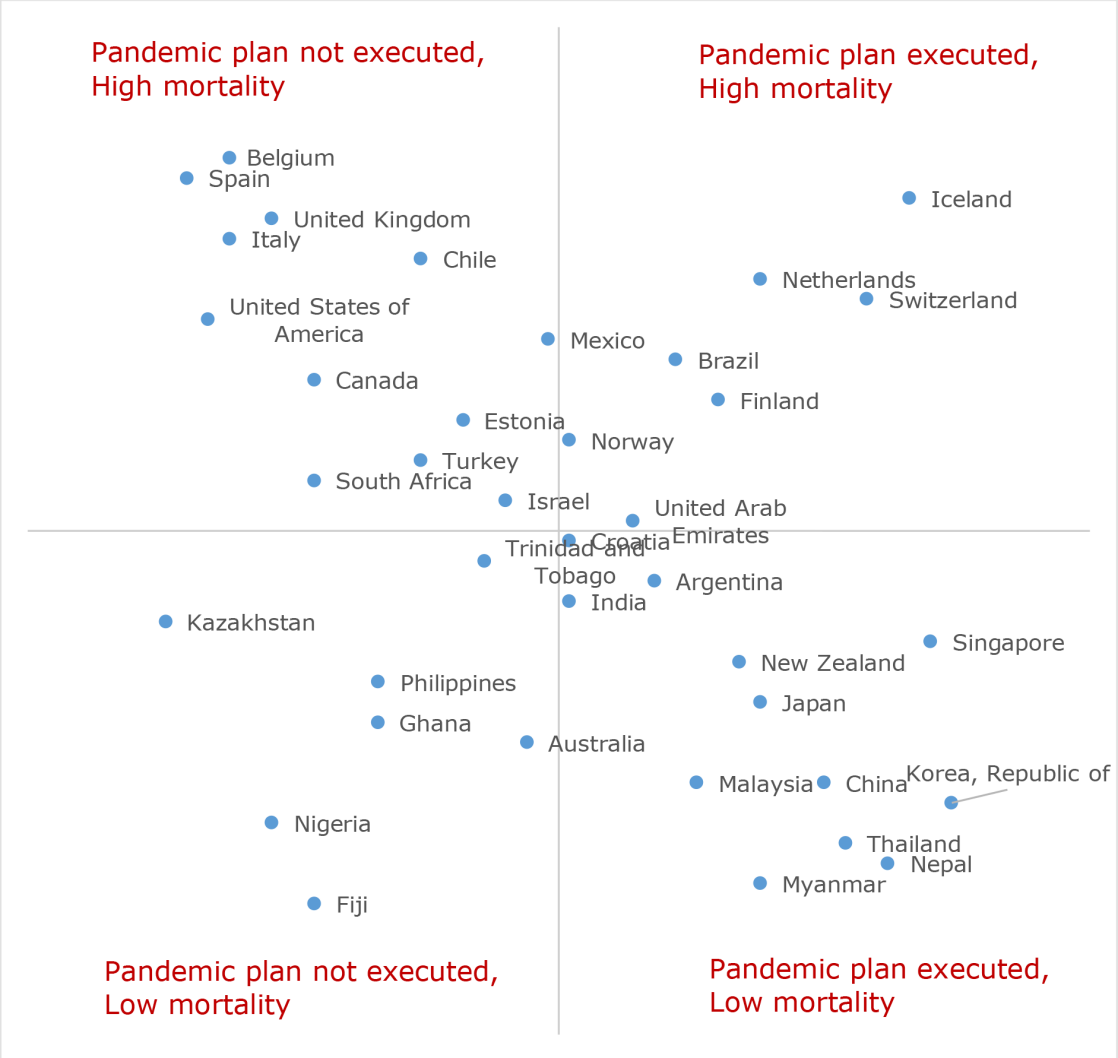
Relationship between pandemic plan execution and maximum COVID-19 mortality rate

A positive correlation was found between the extent to which responders felt a national pandemic strategic plan existed and was executed, and the relevant country’s COVID-19 mortality (Figure 3). There were few notable outlier countries where responders felt a national pandemic strategic plan existed and was executed, yet experienced high mortality; and those who felt a national pandemic strategic plan existed but was not executed, yet experienced low mortality.

### Border closures

**Figure 4.**
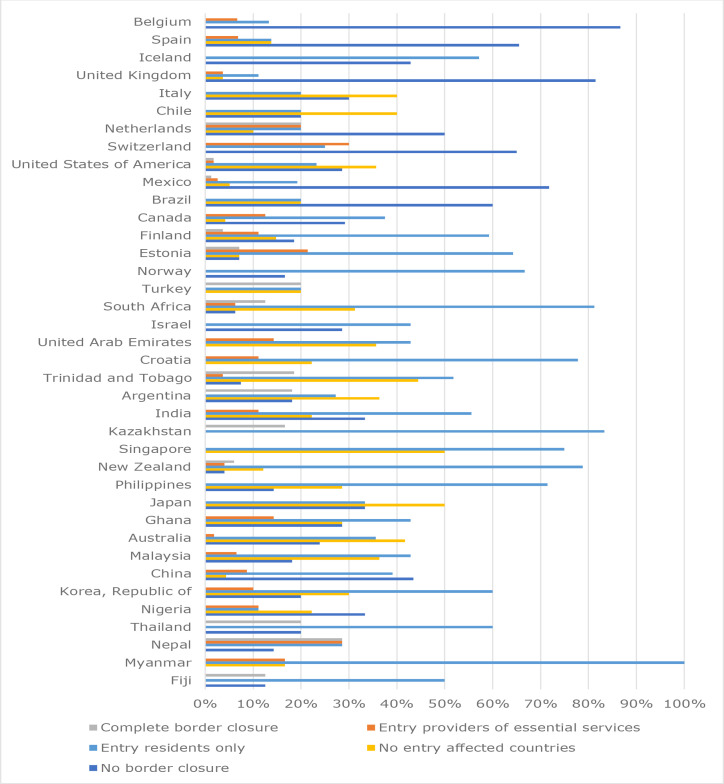
Responses on border closure at time of first mortality for 38 countries from highest (Belgium) to lowest (Fiji) mortality rate

The average of the country-level-aggregated responses across the 38 countries indicating the degree of border closure at the time of first COVID-19 mortality were: no border closures at time of first mortality (26%); no entry for individuals from affected countries (19%); entry only for country citizens and residents (43%); entry only for individuals providing essential services (7%); and complete border closure (5%). (Figure 4). There was a positive correlation with increased mortality for countries where no border closures were applied (*ρ* = 0.47) (see Supplementary Table S3).

### Movement restrictions

**Figure 5.**
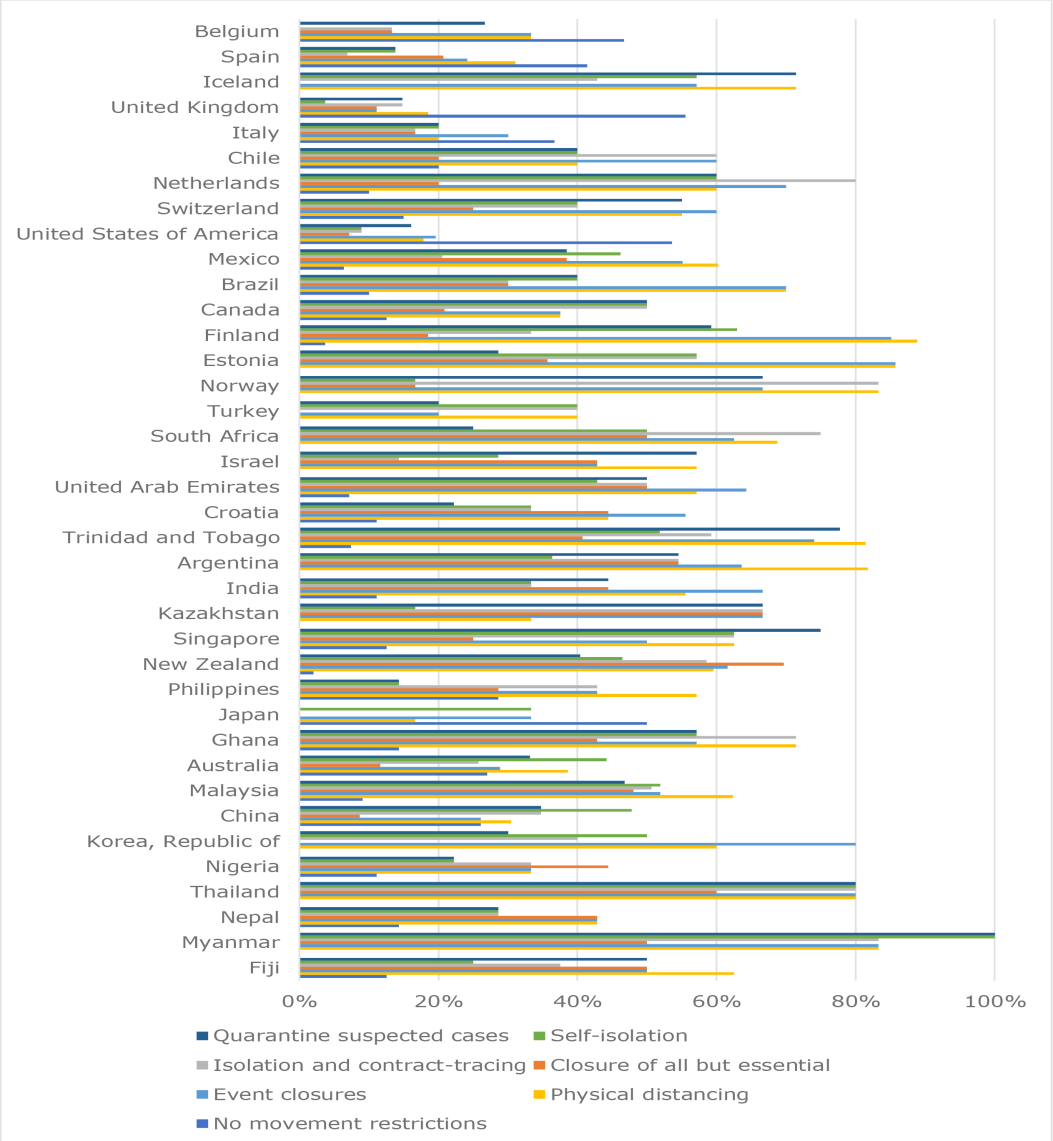
Responses on movement restriction at time of first mortality for 38 countries from highest (Belgium) to lowest (Fiji) mortality rate

The average of the country-level-aggregated responses on the degree of movement restrictions at the time of first COVID-19 mortality were: physical distancing (54%); event closures (53%); self-isolation (40%); selective isolation based on contact tracing (43%); quarantine of suspected cases (43%); and closure of all but essential services (31%). While all 38 countries indicated various strategies in combination at the time of first mortality, closure of all but essential services was selected less compared to other responses (Figure 5).

Bivariate analyses showed negative correlations with mortality for countries where responders believed that movement restriction measures were imposed (*ρ* = -0.15 to -0.44), and a positive correlation with mortality (Pearson's correlation coefficient [*r*] = 0.20) where responders indicated no movement restrictions were imposed (see Supplementary Table S3).

## Discussion

### Summary

These results show that perceived strength of a country’s PC system is not related to early mortality rates from COVID-19. Contrary to the authors’ hypothesis, there was a positive correlation between increased mortality and countries where responders indicated strong PC. High-income countries appear to have been most negatively impacted by COVID-19 mortalities. Countries where responders felt a national pandemic strategic plan existed and was executed experienced lower COVID-19 mortality. Perceived stringent border control, movement restrictions, and testing policies were associated with reduced mortality.

### Strengths and limitations

A strength of this international study is the sample of >1000 PC expert responders from a wide range of countries, who shared their perceptions of their country-level pandemic response. Convenience sampling methods prevent the ability to comment on the representativeness of responders, as well as the ability to report on all nations. The authors attempted to overcome these limitations by eliminating countries from the analysis with <5 responses.

The relationship with mortality rates was chosen as the data around these were likely to be more accurate. Infection rates (number of cases identified) are dependent on who and how many of the population are tested. Countries conducting few tests may appear to have considerably less cases than in reality. Mortality rates may also be under-reported (mortalities at home or in rest-homes may not be counted, for example), but mortality from COVID-19 is likely to have less under-reporting than infection rates.

The present study captured perceptions of PC experts (clinicians, academics, and policymakers) on the strength of their PC system, and their country’s pandemic preparedness and response through survey responses. The authors recognise the limitations of this approach and explored the use of existing objective measures to verify findings. Neither the World Health Organization (WHO) nor the Organisation for Economic Co-operation and Development PHC expenditure datasets cover all 38 countries in the present study on national PHC spending. Currently available surveys of PC strength, such as the Primary Health Care Performance Initiative Trailblazer countries with Vital Signs Profiles, the Primary Health Care Activity Monitor for Europe, and the Primary Care Assessment Tool, are also limited in their scope of countries.

Responders were asked the degree to which they used available data to complete the survey, and how confident they felt about their responses. There was not a strong correlation between these two variables, and it was decided that this analysis added little to the article while increasing its length.

Several confounding factors not addressed may impact interpretation of results. These include whether there are land borders with other infected countries, population density, proportion of population with underlying conditions,^10^ and a high flow of people across borders by tourists,^11^ migrant workers,^12^ or refugees.^13,14^

A multivariate approach was initially envisaged, but unfortunately the nature of the data did not support the approach for the following reasons:

- Nature of the sample*:* regression is a form of inferential analysis, but the present sample is very biased. A descriptive analysis of the findings is more appropriate in this context.
- Sample size: since the dependent variable is at a country level, survey responses must be aggregated to the country level to perform multivariate analyses. To estimate country-level responses with any degree of reliability, multiple responses per country were required; however, ≥10 responses were received for only 21 countries, and ≥5 for 38 countries. This means that a model with two to four predictors at most was possible.
- Multicollinearity*:* there were high levels of collinearity between PC strength variables and other control variables, such as income, population age, and country income.
- High variance*:* there is a high degree of variance in the dependent variable and within-country variance in the predictors. This is problematic given the small sample size.

The above points result in unreliable models, where adding and controlling for additional variables would result in large changes to (previously statistically significant) parameter coefficients. While it was possible to construct an overfitted parsimonious multivariate model with a high *R* ^2^ from a limited number of predictors, given the issues described, that would not have been an honest reflection of the data.

Finally, the present study took place early in the COVID-19 outbreak. The pandemic is still evolving with each country on its own trajectory, hence the recorded maximum mortality rate is dynamic. The availability of only English and Spanish surveys creates bias towards responders with fluency in either. Country selection was further biased by the network dissemination and snowball sampling employed, but mitigated by the early inclusion of leading international organisations’ PC networks (for example, WHO and the World Organization of Family Doctors) in the dissemination strategy.

### Comparison with the existing literature

Growing evidence suggests that country strategy choices were essential if they lacked secondary options.^15^ A country’s health finances determine their capacity for pandemic response, but hospital beds, intensive care units, and trained staff are the big-ticket items. Rapid and stringent border control measures have demonstrated to be effective measures to reduce the speed of the global spread of COVID-19.^16,17^ Where entry of COVID-19 has been prevented through border control, such as in some small Pacific Island nations, morbidity and mortality remain low despite a paucity of healthcare resources.^18^ As relatively high-traffic travel destinations,^11^ the European countries of Italy, Spain, and the UK were rapidly overwhelmed by escalating cases. Border closures and lockdown are drastic measures that have significant social and economic repercussions, including widespread unemployment. While most countries opted to flatten the transmission curve and reduce the rate of mortality, some were more willing to let the infection run its course, aiming to develop herd immunity while minimising social disruption.^19^

The national PHC response — including instigating comprehensive testing at the time of the first mortality and reducing person-to-person contact through hygiene measures and physical distancing, in addition to event and non-essential service closure, self-isolation, and quarantine — appears to have a significant impact on mortality.^17^ Countries with a high mortality rate, such as the UK and US, had a reduced and slower response compared to those with a lower mortality rate, such as Australia and New Zealand.^18^

The COVID-19 mortality rate is disproportionately high in the older population^20^ and people with underlying health conditions.^21^ Countries with high numbers in these groups may have greater mortality rates and need targeted strategies. Reducing physical contact where people live in overcrowded conditions, especially with poor fresh water and sanitation provisions, is additionally challenging.

Clear communication plays a crucial part in national PHC response.^22^ Risk communication needs to provide accessible, consistent, open, and timely information. Where the public are being asked to make big changes in their lives, if they are going to comply they need to understand exactly what they are being asked to do, trust the authority delivering the strategy, and believe that it will make a positive difference to outcomes. Similarly, healthcare workers need to receive clear, accurate, and regularly updated information about service delivery, resource availability, and other key factors in a rapidly changing environment. Strong coherent leadership is key. Further, responding to the extraordinary demands of a pandemic requires communication and integration across a number of sectors: not only between primary and secondary care, but also between the public and private sectors; between public health and PC; and between PC and other community-based health and social services.

A study of the composition of COVID-19 taskforces in 24 countries (including the UK, US, Belgium, Spain, Italy, and Switzerland; all countries in the present study with high mortality rates) found a general absence of PHC, community, and civil society representation in their national government decision making and its response efforts.^23^ Australia, which has had a generally good COVID-19 outcome to date, mounted a medical-driven response, which integrated public health, PC, and community-based services, with clear and consistent communication to both the health workforce and the general population, aimed both at mitigating spread and providing equitable care.^24^

### Implications for practice

Countries perceived by the PC expert responders as having a prepared pandemic plan and a strong PC system did not necessarily experience lower COVID-19 mortality rates; what appears to make a difference to containment of the virus is if and when the plan is implemented, and the degree to which PHC is mobilised to respond. Effective PHC responses including hygiene measures, physical distancing, testing, triaging, and contact tracing, as well as isolation strategies, lead to lower mortality from COVID-19. Public health and PC measures need to be integrated in the PHC response to a pandemic. Lack of correlation with measures of self-reported PC strength suggest poor coordination between PC and public health.

Population lockdown leads to delayed treatment for non-COVID-19 conditions in many patients, with serious health consequences.^25,26^ The disruption to people’s lives leads to increased mental health issues such as anxiety and depression,^27,28^ and dire social consequences for many who have lost their employment. Having strong PC may play an important long-term role in both COVID-19 and non-COVID-19 mortality. Findings from this study can inform policymakers and planners to guide ongoing response to the waves of pandemic response that remain, and inform future preparedness.
